# Blockage of Heme Oxygenase-1 Abrogates the Protective Effect of Regulatory T Cells on Murine Pregnancy and Promotes the Maturation of Dendritic Cells

**DOI:** 10.1371/journal.pone.0042301

**Published:** 2012-08-10

**Authors:** Anne Schumacher, Paul Ojiambo Wafula, Ana Teles, Tarek El-Mousleh, Nadja Linzke, Maria Laura Zenclussen, Stefanie Langwisch, Kristina Heinze, Ivonne Wollenberg, Pablo Ariel Casalis, Hans-Dieter Volk, Stefan Fest, Ana Claudia Zenclussen

**Affiliations:** 1 Department of Experimental Obstetrics and Gynaecology, Medical Faculty, Otto-von Guericke University of Magdeburg, Magdeburg, Germany; 2 Institute of Medical Immunology, Charité, Medical University Berlin, Berlin, Germany; 3 Paediatric Oncology, Hematology and Haemostaseology, Universitätsklinikum Leipzig, Leipzig, Germany; MRC National Institute for Medical Research, United Kingdom

## Abstract

Regulatory T cells (Treg) play an important role in fetal protection. They expand during normal pregnancy and protect fetal antigens from maternal effector cells. Their effect is associated with the up-regulation of tolerance-associated molecules at the fetal-maternal interface. Among these, Heme Oxygenase-1 (HO-1, coded by *Hmox1*) is of special importance as its blockage correlates with increased abortion rates and its up-regulation positively affects pregnancy outcome. Here, we aimed to investigate whether the protective effect of Treg is mediated by HO-1 in a mouse model. HO-1 blockage by Zinc Protoporhyrin (ZnPPIX) abrogated the protective effect of Treg transfer. We found that HO-1 is important in maintaining maternal dendritic cells (DCs) in an immature state, which contributes to the expansion of the peripheral Treg population. This brings to light one essential pathway through which Treg mediates the semi-allogeneic fetus tolerance.

## Introduction

The survival of a developing fetus is a complex process whose success is achieved with stringent tolerance of maternal immune system towards the allogeneic paternal antigens expressed by fetal structures such as the placenta [1]. Tolerance failure may result in immunological pregnancy complications, namely spontaneous abortion or pre-eclampsia [2], [3]. The mechanisms behind the survival of the fetus during gestation are being actively investigated. Some of current theories as to how the maternal immune system actively tolerates the fetus include fetal tissue depletion of tryptophan, an essential amino acid necessary for rapidly dividing cells thereby hindering T cell proliferation [4], expression of human leukocyte antigen G (HLA-G) which blocks the activation of natural killer cells [5], a shift to a Th2 cytokine profile [6] and apoptosis of maternal activated lymphocytes due to the trophoblastic expression of Fas ligand [7]. However, none of these alone can explain fetal survival as IDO [8], FAS [9] and IL-10/IL-4 [10] as well as IL-4/IL-5/IL-9/IL-13 combined [11] knockout mice have normal pregnancy outcome. Recently, a special subset of T cells, regulatory T cells (Treg) [12] has been revelead as important for the survival, acceptance and immune tolerance of developing fetuses [13]–[16].

Successful human and murine pregnancies are clearly associated with an increase in Treg frequency whereas diminished number and function of these cells results in abortion in mice and is associated with miscarriage in humans [13]–[17]. Treg treatment using cells exclusively from normal pregnant mice (CBA/J×BALB/c) was found to completely prevent fetal loss in the abortion-prone murine combination CBA/J×DBA/2J [13], [17], [18]. Treg act by secreting IL-10 [17] and contribute to the generation of a privileged tolerant microenvironment at the fetal-maternal interface, characterized by expression of Heme oxygenase-1 (HO-1), Leukaemia Inhibitory Factor (LIF) and Transforming Growth Factor ß (TGF-ß) [18].

HO-1, a microsomal enzyme involved in the rate-limiting step in the degradation of heme to biliverdin, has been found to be protective in many disease models through its anti-inflammatory, anti-apoptotic and anti-proliferative actions [19], [20]. This enzyme allows acceptance of mouse allograft while its down-regulation results in acute rejection [21], [22]. Furthermore, successful xenograft transplantation is attributed to activation of non-inflammatory protective genes including HO-1 [23]. Absence of HO-1 expression/activity leads to intrauterine fetal death [24] and mating of heterozygote *Hmox1* mice leads to around 6% knockout progeny instead of the expected 25% as for Mendelian rules [25], [26]. We have shown that HO-1 up-regulation by Cobalt Protoporphyrin IX (CoPPIX) as well as by gene therapy results in fetal protection [27], [28]. Novel data links HO-1 and Treg pathways as induction of HO-1 in combination with Donor Specific Transfusion (DST) resulted in successful cardiac transplantation by boosting CD4^+^CD25^+^ T cells [22].

The aim of the present study was to analyze whether the protective effect of Treg in the CBA/J×DBA/2J abortion model is mediated by HO-1. Our data indicate that HO-1 blockage abrogates the protective effect of Treg and provokes abortion. Moreover, blocking HO-1 in Treg donors prevented the ability of these cells to rescue from abortion. We were also able to show that HO-1 blockage renders dendritic cells (DCs) to a mature state that in turn promotes the action of effector T cells (Teff). Accordingly, *in vivo* HO-1 augmentation by CoPPIX keeps DCs in an immature state. This facilitates the expansion and action of Treg. All together, our data demonstrated the importance of the interplay between HO-1 and Treg for maternal tolerance towards the allogeneic fetus.

## Materials and Methods

### Animals

Wild type mice strains of BALB/c and DBA/2J males as well as CBA/J and C57/BL6 females (Charles River and Harlan Winkelmann, Germany) were used. The well-established abortion-prone (AP) combination consisting of CBA/J females mated with DBA/2J males as well as controls having normal pregnancies (NP), CBA/J females mated with BALB/c males, were employed in this study [29]. *Foxp3^gfp^* transgenic mice in a C57/BL6 background [30], kindly provided by Prof. Rudensky, were also included in the study. *Hmox1* ^+/+^, *Hmox1* ^+/−^ and *Hmox1* ^−/−^ mice (in BALB/c background) kindly provided by Dr. Miguel Soares and Dr. S-F Yet [26], were used to study maturation of isolated DCs as well as their cytokine secretion after 24 h of culture in medium with or without the addition of LPS. All animals were maintained in our animal facility. The animals were treated according to the institutional guidelines with the ministerial approval (LaGeSo 0070/03 and Landesverwaltungsamt Sachsen-Anhalt AZ2/868) and the experiments were conducted by authorized persons according to the Guide for care and use of animals in Agricultural research and teaching. The animals were checked twice a day for vaginal plugs (8 AM and 7 to 9 PM) and the day of plug detection was considered to be day 0 of the pregnancy.

### Experimental setting

To understand the participation of HO-1 in Treg-mediated tolerance towards the fetus we carried out the following series of experiments:

- We first blocked HO-1 in NP and AP animals by means of ZnPPIX. The same was done with AP animals that additionally received Treg (NP animals on day 14 served as donors). ZnPPIX (Frontier Scientific, Logan, Utah, USA) was applied intraperitoneally (i.p.) at 40 mg/kg concentration. Controls received PBS (both in 100 µl final volume). Injections were done on days 0, 3 and 6 of pregnancy. In further experiments we transferred Treg from donor animals that have been previously treated with ZnPPIX using the same schema. Adoptive Treg transfer was achieved on day 1 of pregnancy [13]. Animals were killed on day 14 of pregnancy. Groups consisted of 5–22 animals.
- We employed *Foxp3^gfp^* transgenic allo-pregnant animals to understand to which extent Treg number or functions are affected after HO-1 down-regulation. For this we treated *Foxp3^gfp^* pregnant females previously mated with BALB/c animals with either PBS (100 µl) or ZnPPIX (40 mg/kg) i.p. on days 0, 2 and 4 of pregnancy. Females were sacrificed at day 5 and the levels of Foxp3^+^GFP^+^ cells were analyzed by flow cytometry. For analyzing Treg functionality, the cells were first stained for CD4 and then GFP^+^ cells (Foxp3 expressing cells) within this population were sorted using FACS Diva Flow Cytometry and Cell Sorter (Biosciences, Franklin Lakes, New Jersey, USA). Sorted cells were used for proliferation assays in which PKH26-stained lymphocytes from total lymph nodes (responder cells) obtained from day 5 allo-pregnant BALB/c-mated C57/BL6 wild type females were co-cultured in 1∶1 ratio with sorted cells. Responder cells were harvested at time points 0, 24 and 48 h and their proliferation at different time points was measured using flow cytometry.
- To understand the mechanisms through which HO-1 mediates Treg effects, we studied the influence of HO-1 on DC maturation assuming that immature DCs support Treg expansion. We modulated HO-1 in NP females by either *in vivo* augmenting HO-1 using CoPPIX (Frontier Scientific, Logan, Utah, USA) at a dose of 5 mg/kg or by its blockage using ZnPPIX (40 mg/kg). PBS-treated NP animals served as controls. All treatments were done i.p. on days 0 and 3 of pregnancy and animal preparations were done on day 5 of gestation. We isolated splenic DCs from these animals, which were co-cultured 1∶1 with CD4^+^ responder cells obtained from total lymph nodes of NP females at day 5 of pregnancy that had been stained with CFDA-SE. Cells were then harvested at time points 0, 24 and 48 h. Further experiments consisted of characterizing the maturity markers of DC isolated by MACS-technology from spleen of HO-1 deficient or competent mice, e.g. *Hmox1* ^+/+^, *Hmox1* ^+/−^ or *Hmox1* ^−/−^ female mice and cultured in RPMI medium added of 10% of fetal calf serum, antibiotics and 50 µM of b-mercaptoethanol. LPS was added in a concentration of 1 µg/ml.
- In order to document the changes observed in DCs after HO-1 modulation we isolated DCs from bone marrow of NP animals on day 5 following the method established by Lutz *et al*. [31] and cultured them for 12 days in the presence of 20 ng/ml recombinant mouse GM-CSF (Serotec, Raleigh, NC, USA). Thereafter, DCs were exposed to either LPS (1 µg/ml, Fluka Biochemika, Buchs, Switzerland) alone, LPS+CoPPIX (50 µM) or LPS+ZnPPIX (20 µM) for 48 h. DCs were later characterized for the expression of maturation markers, CD11CD80 and CD11MHCII on their cell surface by flow cytometry as well as for their cytokine secretion (IL-10 and TGF-ß, IFN-γ) in culture supernatant after 48 h.

Exact number of animals for each experiment is indicated in Figure legends.

### CD4^+^CD25^+^ Treg, responder cells and DC isolation

Treg were isolated from spleen and thymus as previously described [13] and 2×10^5^ (200 µl) of these cells were injected intravenously (i.v.) to recipient mice on day 1 of pregnancy or used for *in vitro* studies. CD4^+^ T cells (responder cells) were isolated from either total lymph nodes or draining lymph nodes of pregnant mice using the CD4^+^T isolation kit (Miltenyi Biotec GmbH, Bergisch Gladbach, Germany) and were stained with CFDA-SE using the standard procedure [16]. Splenic DCs were isolated from differently treated NP females (either with PBS, Zn-PP or Co-PP) using CD11c isolation kit (Miltenyi Biotec GmbH, Bergisch Gladbach, Germany). Bone marrow-derived DCs were isolated and cultured as described in [31].

### Animal preparation and tissue harvest

Blood, decidua, placenta, thymus, spleen and either total or local lymph nodes were obtained as described elsewhere [13]. Samples were either immediately processed for flow cytometry, snap frozen and kept at −80°C for RNA and protein isolation or fixed in ethanol 96% for later embedding in paraffin as described elsewhere [32].

### Flow Cytometry

Cells were isolated from spleen, blood, lymph nodes, uterus or decidua [15] and stained with following antibodies: FITC-conjugated rat anti-mouse CD4 (clone: RM4-4), Cy5-conjugated rat anti-mouse CD8 (clone: 53-6.7) and either PE labeled anti-mouse CD25 (spleen samples, clone: PC61) or PE-labeled anti-mouse Foxp3 (all other tissues, clone: NRRF-30). Uterine and blood cells were stained with the following antibodies: APC-labeled anti-mouse CD11c (clone: HL3), PE- anti-mouse I-A/I-E (MHC II, clone: M5/114.15.2)), PE-Cy7 Hamster anti-mouse CD69 (clone: H1.2F3) and PE-anti mouse CD80 (16-10A1)..Unstained cells or cells stained with isotype controls served as controls for the gating and quadrant setting. All antibodies were purchased from Becton Dickinson, Heidelberg, Germany besides Foxp3 antibody, which was from E-Bioscience, Kranenburg, Germany. Samples were analyzed using a FACS Calibur (BD Biosciences, Franklin Lakes, New Jersey, USA).

### RNA isolation and Real-time RT-PCR

Total RNA was isolated from uterine, placental or decidual tissue by means of TRIzol method (Invitrogen Life Technologies, Paisley, UK) using a homogenizer (Ultra-Turrax T25; Labortechnik, Staufen, Germany) [13]. RNA was resuspended in RNase-free water (Braun) to a final concentration of 1 µg/µl and kept at −80°C until use. 2 µg of RNA was used for cDNA synthesis as described before [13]. For real-time PCR analysis, 2 µl cDNA were used as starting volume to amplify the DNA. PCR-Mastermix (6.25 µl; Eurogentec), 3 µl of the primer mix, 0.5 µl of the fluorescent probes, and RNase-free water were added to a final volume of 13 µl. The amplification reactions were performed on the ABI Prism 7700 Sequence Detection System (PerkinElmer Applied Biosystems, Darmstadt, Germany) as follows: 95°C for 10 min, followed by 95°C for 15 s and 1 min at 60°C in a total of 60 cycles before a final cycle at 50°C. β-actin was used as housekeeping gene. The expression of mRNA was calculated as 2^−ΔCt^. Primers and probe sequences are provided in Table S1.

### SDS-polyacrylamide gel electrophoresis and Western blot

Proteins were extracted from frozen samples as described elsewhere. 50 µg of protein were used for SDS-polyacrylamide gel electrophoresis separated on a 10% running gel at 110 V. Proteins were then transferred to nitrocellulose membranes (BioRad) at 4°C (180 mA). After an overnight (ON) incubation with 5% milk powder in TBS and washing with a solution containing 0.05% Tween 20 and 5% milk powder in PBS, the membranes were incubated first with the primary antibodies, rabbit anti-HO-1 (StressGen, Victoria, British Colombia, Canada; 1∶1000) and GAPDH (Santa Cruz Biotechnology, Santa Cruz, USA; 1∶1000) for 2 h at room temperature (RT) with agitation and after washing, they were incubated with secondary antibody, polyclonal anti-rabbit (DAKO) for 1 h at RT with agitation. Membranes were incubated with an Avidin/Biotin complex for 30 min at RT, the bands were visualized by chemoluminescence (ECL substrate from Amersham, Germany) and exposed onto Kodak Miomax MR Imaging film (Sigma, Cedex, France). The intensity of the bands was quantified on a GS-800 calibrated densitometer (Biorad, Germany) and the individual values were obtained using a computer software program (Quantity One; BioRad, Hercules, CA, USA).

### Proliferation assays

Proliferation assays were performed by co-culturing CD4^+^Foxp3^+^ cells from *Foxp3^gfp^* animals and responder cells from wild type animals. Foxp3^+^ cells were sorted from total lymph nodes of *Foxp3^gfp^* mice previously mated with BALB/c at day 5 of pregnancy after the mice were treated either with ZnPPIX (40 mg/Kg) or PBS (100 µl). Responder cells were isolated from pregnant BALB/c-mated C57/BL6 females (day 5) and stained with PKH 26 red fluorescent dye (Sigma-Aldrich Chemie, Steinheim, Germany). Foxp3^+^ cells were co-cultured with responder cells in a ratio of 1∶1 in RPMI+10% FBS medium in 96-well plates. Responder cells were harvested at time points 0, 24 and 48 h and their proliferation was measured using flow cytometry. The experiments were performed six times and each assay was done at least in duplicates.

In a second setting, splenic DCs were isolated from NP females (day 5) using microbeads (Miltenyi Biotec GmbH, Bergisch Gladbach, Germany) after initially digesting with Collagenase D (Roche Diagnostic, Penzberg, Germany). HO-1 levels had been previously *in vivo* modulated by CoPPIX or ZnPPIX as described before. NP animals treated with PBS served as controls. Treatments were performed on days 0 and 3 of pregnancy and animals were killed on day 5 of gestation. CD4^+^ responder cells obtained from total lymph nodes of NP females were stained with CFDA-SE. Spenic C11c^+^ DCs were co-cultured with responder cells in a ratio of 1∶1 in 96-well plates. Cells were then harvested at time points 0, 24 and 48 h and were analyzed for the proliferation of responder cells.

### ELISAs

Supernatants were harvested from bone-marrow derived DCs which had been exposed to LPS (1 µg/ml) alone, LPS+CoPPIX (50 µM) or LPS+ZnPPIX (20 µM) for 48 h. Anti-inflammatory cytokines TGF-ß1 (ebioscience, San Diego, CA, USA) IL-10 (BD, Bioscience, San Jose, CA, USA) as well as the pro-inflammatory cytokines IL-6 and IL-12 (BD, Bioscience, San Jose, CA, USA) were measured following protocols provided by the manufactures. In brief, 96-well microwell tubes (Nunc, Roskilde, Denmark) were coated with 100 µl of capture antibodies at 1∶250 dilution ON at 4°C. After washing, the plates were blocked for 1 h using assay diluent at RT. Alongside standards of respective cytokines, samples were loaded in duplicates and were incubated for 2 h at RT. Washing followed to remove unbound antigens and primary detection antibodies which were linked to Streptavidin-Horse Radish Peroxidase. Incubation with the antibodies was done for 1 h at RT and a substrate was added after washing. The reaction was stopped using 1 N sulphuric acid and absorbance was read at the recommended wavelength within 30 min.

### Statistical analysis

Because of the not normal distribution of the *in vivo* data, statistical analysis was performed using the non-parametric Kruskal-Wallis test followed by Mann-Whitney-*U* test. In all cases, *p*<0.05 was considered a statistically significant difference. Accordingly, *in vivo* data are presented as median values. Statistical analysis for proliferation assay was done by Dunn's multiple comparison test and the results of six experiments are presented as means. Correlation analysis was performed using Pearson's Test. Tests used and number of samples (n) for each experiment is indicated in the Figure legends.

## Results

### Hmox1 and foxp3 mRNA levels in murine uterine tissue correlate positively, while low HO-1 protein levels are associated with augmented abortion rates

**Figure 1:**
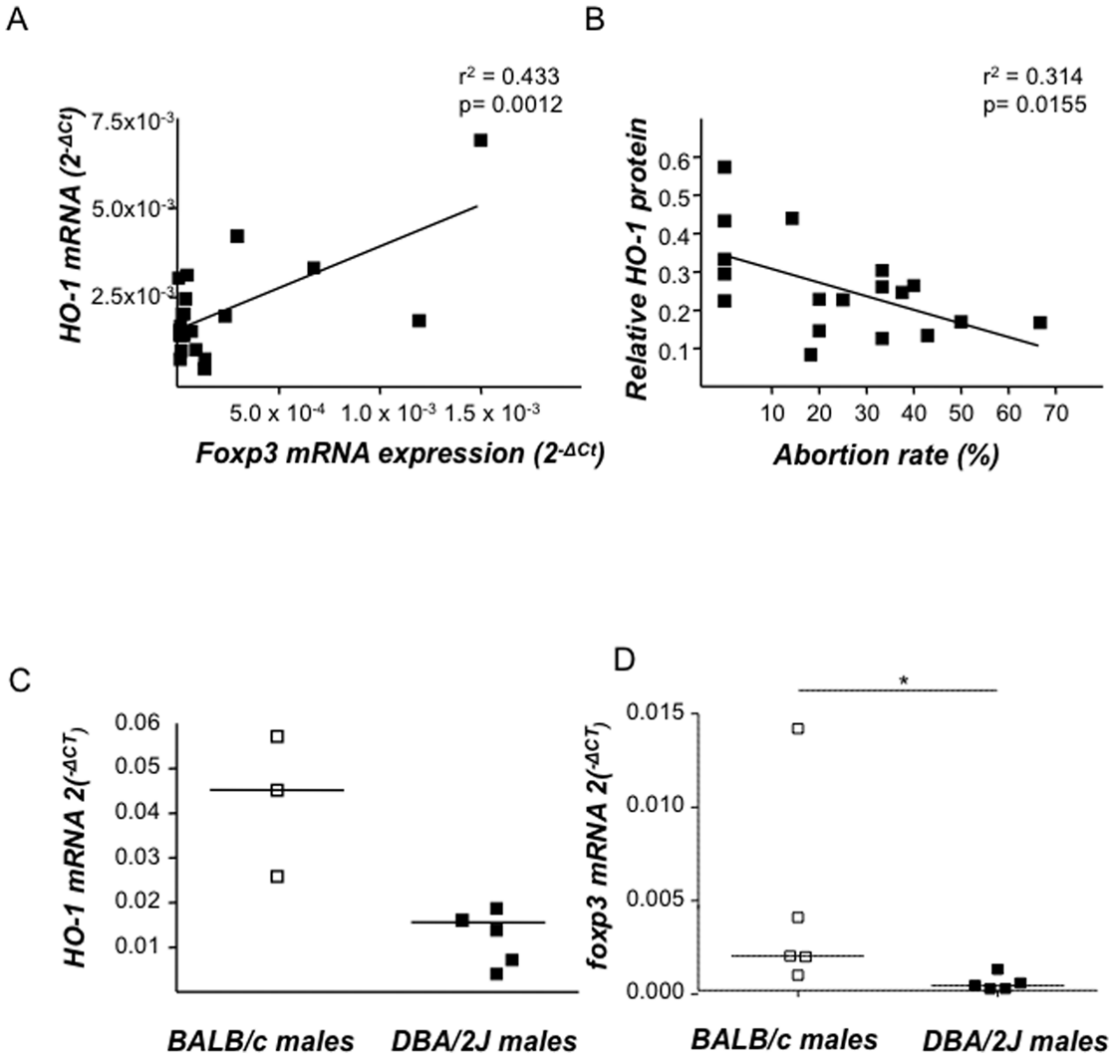
Hmox1 and foxp3 mRNA expression positively correlate in pregnancy and HO-1 protein levels determine pregnancy outcome. **A:** The correlation between Hmox1 and foxp3 mRNA levels in uterus of normal pregnant specimens was analyzed. The samples used in this study were uterine, decidual and placental tissues (n = 21) obtained from day 0 until 8 of pregnancy from normal pregnant (NP) females from which mRNA expression of both of *Hmox1* and *foxp3* was analyzed using real time RT PCR. Correlation analysis was performed using Pearson analysis program. r^2^ represents co-efficient of determination value while p indicates the exact levels of significance. **B:** Total protein was obtained from decidual tissue of day 14 pregnant abortion-prone (AP) or NP animals and HO-1 levels were determined by Western Blot. The protein expression intensity level values (n = 18) were obtained from band intensities using Quantity 1 and were correlated to the abortion rates of the respective animals using Pearson's correlation analysis. The sample distribution was tested for normality using KS test. Correlation significances are indicated by p value. **C**: *Hmox1* mRNA levels in testicle samples of BALB/c (n = 3) and DBA/2J males (n = 5) were determined using real time RT-PCR. **D**: *foxp3* mRNA levels in spleen samples of BALB/c (n = 5) and DBA/2J males (n = 5) were determined using real time RT-PCR. Results are presented as median mRNA levels in dot plots. The statistical differences were analyzed using non-parametric Mann-whitney-*U*- test which gave a p value of 0.036 (*p<0.05).

We have previously proposed that HO-1 and Foxp3 levels are both relevant to pregnancy outcome in different models [24], [33], [34]. Here, we examined the levels of *Hmox1* and *foxp3* mRNA in uterus (days 0–5), decidua (days 5–14) and placenta (beginning on day 8 of pregnancy) from NP mice and found that their expression correlate positively (r^2^ = 0.433; p = 0.0012; Fig. 1A). An inverse correlation between foxp3 mRNA and abortion rates in early stages was well-documented [34]. Here we additionally confirmed that low HO-1 protein levels, as it occurs naturally in AP mice, correlate inversely with abortion rates (r^2^ = 0.3134, p = 0.0155), while high HO-1 levels in NP mice are associated with low abortion rates (Fig. 1B). Interestingly, the difference in HO-1 expression in placental tissue between NP and AP mice seems to be dictated by the paternal component, which is in fact, the only genetic difference between both F1 tissues as DBA/2J males present significantly lower *Hmox1* mRNA levels in several tissues when compared to BALB/c males. Fig. 1C shows representatively *Hmox1* mRNA levels in testicles from both male strains while levels of *Hmox1* mRNA for spleen and thymus are shown in Figure S1. Similarly, Foxp3 mRNA levels were diminished in spleen from DBA/2J males compared to BALB/c ones (Fig. 1D).

### In vivo down-regulation of HO-1 by ZnPPIX during pregnancy leads to diminished Treg frequency provokes fetal death in the CBA/J×DBA/2J model

**Figure 2:**
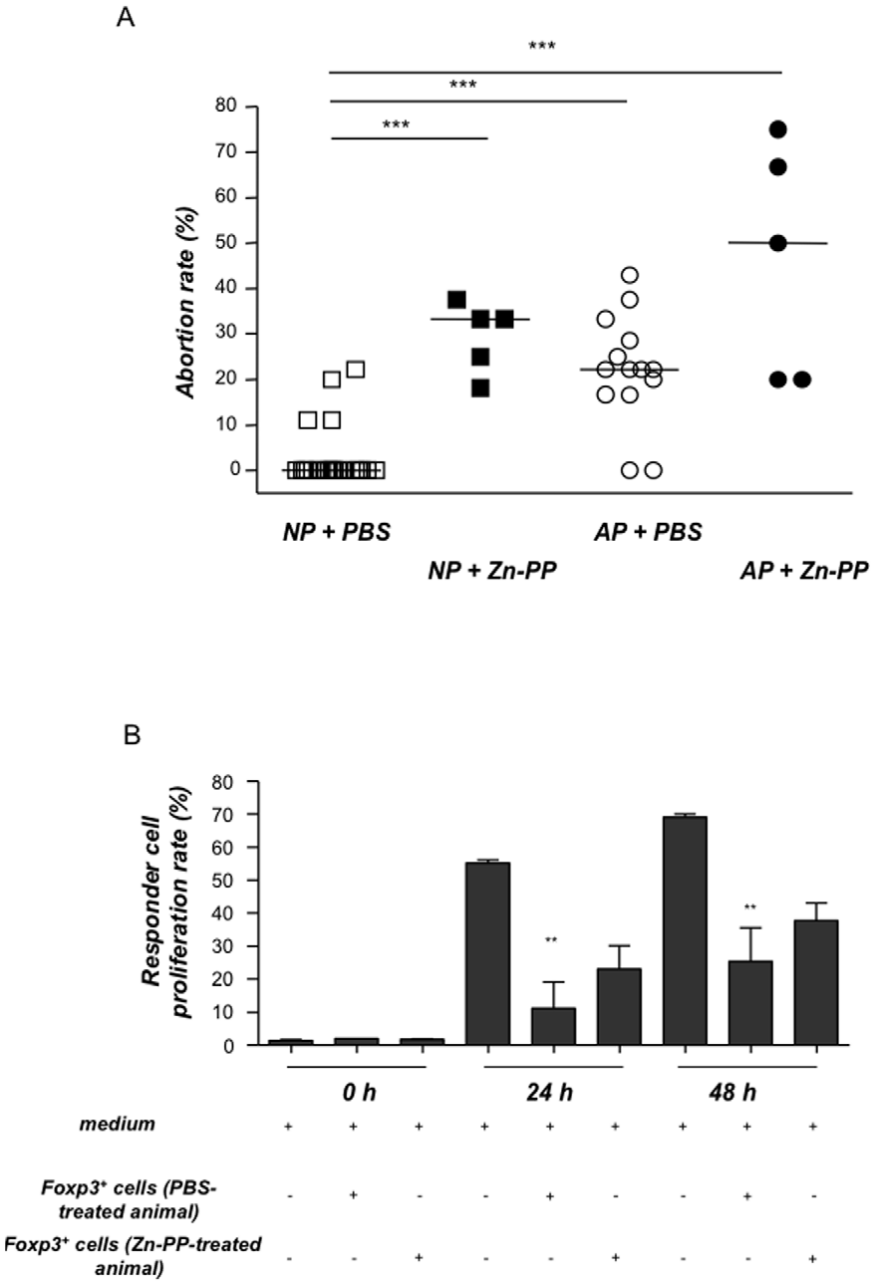
HO-1 blockage results in increased abortion rates and leads to less suppressive CD4^+^Foxp3^+^ cells in vitro. Normal pregnant (NP) or abortion-prone (AP) animals were treated either with PBS (NP = 22, AP = 14) or ZnPPIX at a concentration of 40 mg/kg in 100 µl (n = 5 NP and n = 5 AP). Treatments were done intraperitoneally on days 0, 3 and 6 of pregnancy. Animals were sacrificed on day 14. **A:** Abortion rates were determined on day 14 of pregnancy from NP, AP animals together with their corresponding Zn-PP-treated groups. The results are presented as median abortion rates. The statistical differences were obtained using non-parametric Mann-Whitney-*U*- test and Kruskal Wallis Test. Significant differences in the abortion rates between the controls and Zn-PP-treated groups are shown by p values. ***p<0.001 for all of them. **B:** shows the proliferation rate of responder cells which have been co-cultured with Treg from ZnPPIX- or PBS-treated animals. Responder cells (from lymph nodes) were isolated from day 5-pregnant wild type C57/BL6 females which had been mated with a BALB/c male and further co-cultured with CD4^+^Foxp3^+^GFP^+^ regulatory cells sorted from either ZnPPIX or PBS-treated allo-pregnant *Foxp3^gfp^* mice. The proliferation rate of responder cells was determined using flow cytometry at different time points, namely 0, 24 and 48 h. Data (mean ± SEM) represent percentage of responder cell proliferation of six independent experiments. Statistical analyses were done using one way ANOVA. Significant differences in the proliferation rate of responder T cells between responder T cells alone and responder T cells plus Foxp3+ cells from PBS-treated animals are shown by p values **: p<0.01.

We next investigated whether the *in vivo* down-regulation of HO-1 would have any impact on the levels or on the activity of Treg. For this, we treated NP or AP animals with three ZnPPIX doses that ensure that the effect of ZnPPIX is profound, while control animals from both NP and AP groups received PBS. HO-1 blockage by ZnPPIX provoked a trend towards a decrease in the levels of CD4^+^Foxp3^+^ cells in spleen and blood (data not shown). At the fetal-maternal interface ZnPPIX diminished the percentages of CD4^+^Foxp3^+^ cells as compared to their respective controls (NP+PBS: 1.32%, NP+ZnPPIX: 0.54%, #: p:0.1). Treatment with ZnPPIX increased the median abortion rate from 0% to 33.3% in females from the NP group confirming that the blockage of HO-1 results in fetal rejection (Fig. 2A). ZnPPIX treatment boosted the abortion rate of AP females from 22.2% to 50% (Fig. 2A). ZnPPIX provoked no cytotoxicity as splenomegalia, enhanced lymph nodes, and any other inflammation signs could be discarded. In addition, the application of CoPPIX, a porphyrin, did not have any negative effects on CBA/J×BALB/c pregnancies and was able to significantly diminish the abortion rate in CBA/J×DBA/2J combinations [26]. Our data confirm that HO-1 blockage interferes with the normal development of allogenic pregnancy while probably affecting Treg frequency.

### In vivo HO-1 blockage by ZnPPIX affects the suppressive activity of Foxp3^+^ cells

It is already known that HO-1 deeply affects pregnancy outcome. It is however unknown whether HO-1 changes affect Treg function during pregnancy, as it does in other models of physiological and pathological relevance [20], [22]. To investigate to which extent the Foxp3***^+^*** population is functionally affected by ZnPPIX treatment we employed a second model, taking advantage of the *Foxp3^gfp^* mice [29], whose Foxp3^+^ cells are positive for GFP and can be detected by flow cytometry or purely isolated by cell sorting. We initially analyzed the impact of HO-1 down-regulation on the number of Foxp3^+^ cells in allo-pregnant *Foxp3^gfp^* mice treated with ZnPPIX compared the results to those obtained with the PBS-treated controls. We could observe a diminution in the number of Foxp3^+^ cells in the iliac lymph nodes 1 day after the last ZnPPIX treatment (median: 3.88% vs. 5.01% of Foxp3^+^ cells; we refrained doing statistics because of the low number of animals per group (n = 2/3). Interestingly, the frequency of IL-10 producing Treg was also diminished in these mice after ZnPPIX therapy (Figure S2). We next sorted Foxp3^+^GFP^+^ cells from either ZnPPIX or PBS-treated allo-pregnant *Foxp3^gfp^* mice and co-cultured them with responder cells from pregnant wild type C57/BL6 females. Foxp3^+^ cells were able, as expected, to significantly suppress the proliferation of responder cells. CD4^+^Foxp3^+^ cells from ZnPPIX-treated animals were not longer able to suppress responder cells, indicating that HO-1 blockage affects Treg suppressive function (Fig. 2B).

### Blocking HO-1 by means of ZnPPIX abrogates the protective effect of Treg on pregnancy outcome

**Figure 3:**
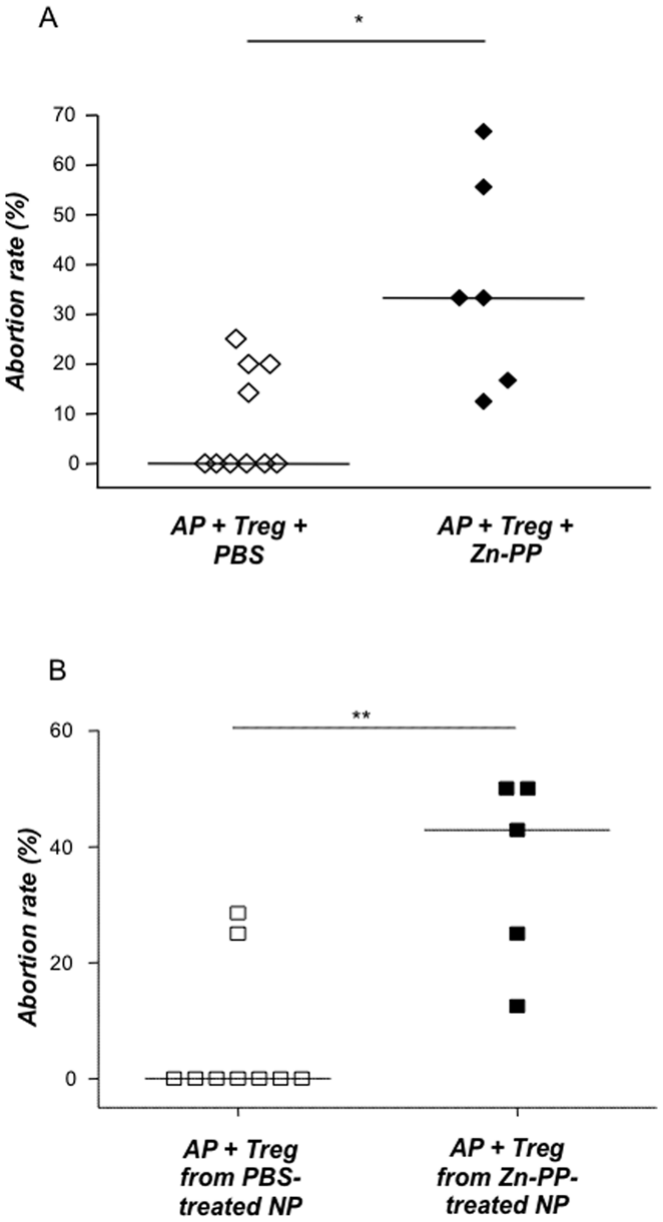
HO-1 blockage abrogates the protective effect of Treg. (**A**); Abortion-prone animals were adoptively transferred with Treg (2×10^5^ cells) isolated from normal pregnant animals treatment known to prevent fetal rejection. The animals received either PBS (100 µl; n = 10) or ZnPPIX (40 mg/kg; n = 6) intraperitoneally on days 0, 3 and 6 of pregnancy and were sacrificed on day 14. (**B**)**:** As in (A) NP animals were transferred with Treg. These Treg, however, derived from either PBS- or ZnPPIX-treated donor mothers. For (A) and (B) Abortion rates were determined on day 14 of pregnancy upon animal preparation and are calculated as a percentage of the ratio of resorptions to total implantation sites. The data is presented as median abortion rates. Statistical differences were analyzed by the non-parametric Mann-Whitney-*U*- test and the statistical difference obtained between the groups was *: p<0.05 and **:p<0.01.

After confirming that HO-1 affects the percentages and functionality of Treg during pregnancy, we next aimed to study to which extent the *in vivo* blockage of HO-1 affects Treg protective effect on pregnancy. We therefore adoptively transferred Treg into abortion-prone females while blocking HO-1 by means of ZnPPIX. Treg-transfer and PBS resulted in total fetal protection as expected (Fig. 3A) [13]. HO-1 blockage by ZnPPIX completely abrogated the protective effect of Treg, resulting in significantly higher abortion rates (33.3%; p = 0.011, Fig. 3). ZnPPIX blockage did not interfere with the implantation rates as both groups showed comparable implantation numbers (data not shown). Ovulatory effect should also be discarded as ZnPPIX treatment was initiated after pregnancy was confirmed. This suggests that the effect of HO-1 blockage relies on Treg not longer preventing effector cells from rejecting the fetuses. To understand whether HO-1 blockage *in vivo* affects Treg quality and functionality we next treated animals that served as Treg donors with ZnPPIX as described, isolated Treg and transferred them into pregnant females [13]. Most interestingly, Treg from ZnPPIX-treated animals were no longer able to protect from abortion (Figure 3B).

### HO-1 contributes to maintain DCs in an immature state and enhances the proliferation of Treg in vitro

**Figure 4:**
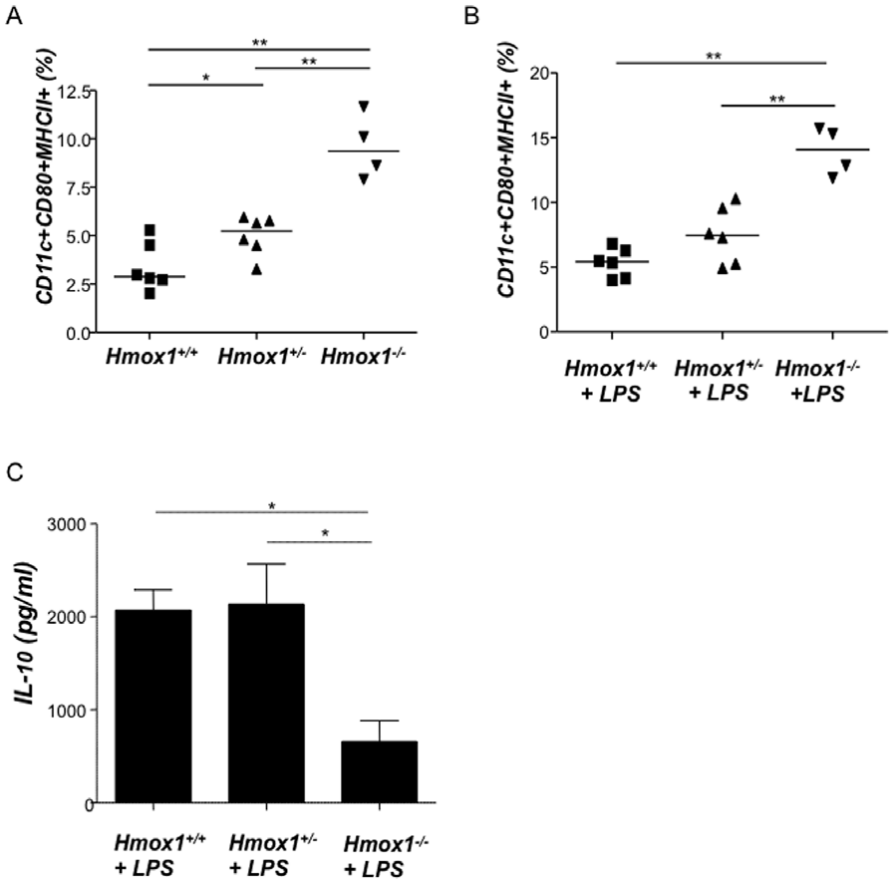
Splenic dendritic cells from Hmox1^+/−^ and Hmox1^−/−^ female mice are more mature than those obtained from Hmox1^+/+^ controls. Dendritic cells (DCs) were isolated from spleen of *Hmox1* ^+/+^ (n = 5), *Hmox1* ^+/−^ (n = 6) or *Hmox1* ^−/−^ (n = 4) females, cultured with (**B**) or without (**A**) LPS addition (1 µg/ml) and analyzed for the expression of maturity markers CD11c, CD80 and MHCII. In (**C**), the IL-10 levels secreted by DCs from these animals were measured by ELISA. Differences among the groups were analyzed by the Mann-Whitney-U test following Kruskall-Wallis test. *:p<0.05 and **:p<0.01.

**Figure 5:**
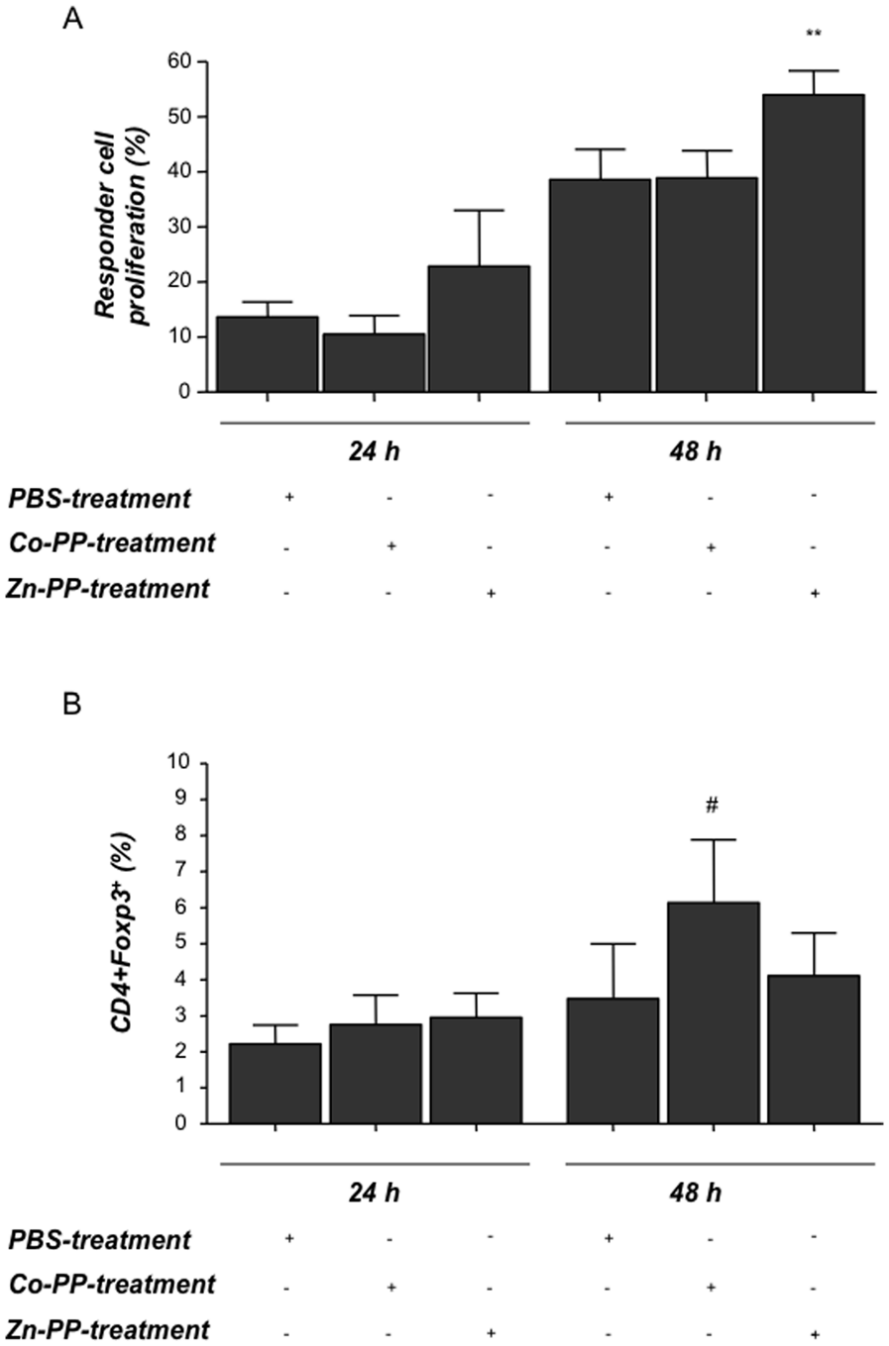
Dendritic cells from ZnPPIX-treated animals stimulate the proliferation of responder cells while dendritic cells from CoPPIX-treated animals influence Foxp3 expression in T cells. Splenic dendritic cells (DCs) from different HO-1 environment (obtained from PBS-, CoPPIX- or ZnPPIX-treated animals) were co-cultured with CFSE-stained CD4^+^ responder cells which were isolated from lymph nodes of NP females (day 5 of pregnancy) in a ratio of 1∶1. Cells were harvested at time points 0, 24 and 48 h and proliferation was measured by flow cytometry. **A** shows the proliferation of responder cells after co-culture with DCs at time points 24 and 48 h, which was calculated with respect to time point 0. The results shown are obtained from six independent experiments. Data is shown as mean ± SEM. Statistical analyses were done using one way ANOVA. **: p<0.01. **B** shows the expression of Foxp3 in responder cells, depicted as percentage, as analyzed by flow cytometry after co-culturing these cells with DCs from animals with different HO-1 environment (24 h and 48 h). The data (mean±SEM) is representative of six independent experiments. Statistical analysis was done using one way ANOVA and # means p<0.1.

It is known that *Hmox1* deficient mice have normal Treg levels [35], however it is possible that their function is suboptimal because other players as DCs are affected by HO-1 deficiency. Chauveau *et al*. first reported that HO-1 interferes with the maturation of DCs [36] in transplantation models. Immature DCs have been shown to produce HO-1 and may be responsible for the inhibition of pro-inflammatory and allogeneic immune response [37]. Thus, we hypothesized that HO-1 up-regulation supports the maintenance of DCs in a rather immature state while HO-1 down-regulation fosters their maturation. Immature or tolerogenic DCs are known to be involved in tolerance [37] including tolerance towards the fetus [38], while mature DCs are rather found in normal immune responses directed to destroy and not to protect foreign antigens [37]. To confirm whether HO-1 modulates DC maturation we moved to a different model and analyzed the percentages of CD11c^+^CD80^+^MHC class II^+^ cells in isolated DCs from wild type, heterozygote and knockout mice for HO-1 in BALB/c background (*Hmox1* ^+/+^, *Hmox1* ^+/−^ and *Hmox1* ^−/−^ mice). We observed that splenic DCs isolated from *Hmox1* ^+/−^ and *Hmox1* ^−/−^ mice and cultured with or without LPS are significantly more mature than those obtained from *Hmox1* ^+/+^ females (Fig. 4A and Fig. 4B respectively). This confirms previous data addressing the role of HO-1 on DC maturity [36], [39], [40], [41]. LPS-stimulated cultured DCs from HO-1 deficient animals were not able to produce as much IL-10 as DCs from HO-1 competent mice did (Fig. 4C), further contributing to the idea of HO-1 inducing tolerogenic DCs. To investigate to which extent HO-1 modulation during pregnancy affects DC functionality, we isolated splenic DCs from pregnant females with normal (+PBS), high (+CoPPIX) or low (+ZnPPIX) HO-1 levels and put them in culture with CD4^+^ T effector cells isolated from total lymph nodes of untreated NP female mice on day 5 of pregnancy (henceforth referred to as responder cells, these are primed to paternal antigens). Responder cells showed higher proliferation after being in culture with DCs obtained from ZnPPIX treated females (low HO-1) than when in contact with DCs from PBS-treated controls (Fig. 5A). Our data suggests that low HO-1 enables the existence of a fully functional mature DC population (mDCs) which is then able to present paternal antigens, hence facilitates the proliferation of responder/effector cells. CoPPIX-treated DCs, on the contrary, did not boost the proliferation of responder cells but rather favoured DCs to remain in an immature state as they already are in pregnant state (Fig. 5A). Immature DCs (iDCs) are known to be tolerogenic [41], hence shift the immune response towards tolerance in which foreign antigens (such as paternal antigens during pregnancy) are more actively tolerated than rejected. When characterizing the phenotype of CD4^+^ responder cells after the co-culture, we observed that the contact of responder cells with DCs from CoPPIX-treated mice resulted in a transient augmentation of Foxp3 expression in CD4^+^ cells as compared to cells co-cultured with DCs from the PBS or ZnPPIX treated counterparts (Fig. 5B). Our results strongly suggest that HO-1 contributes to the maintenance of DCs in an immature state hence renders them tolerogenic, thus facilitates the expansion of peripheral Treg and helps in the suppressive effect of Treg.

### HO-1 blockage promotes DC maturation while HO-1 up-regulation provokes the secretion of anti-inflammatory cytokines, IL-10 and TGF-ß while diminishing the IL-12 secretion by DCs in vitro

**Figure 6:**
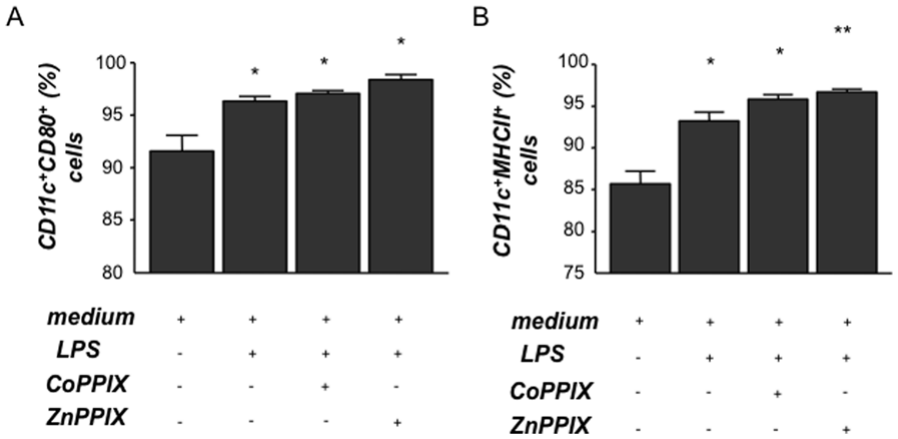
In vitro HO-1 down- regulation by ZnPPIX treatment results in more mature bone-marrow-derived DCs. Bone-marrow-derived dendritic cells (DCs) were isolated from normal pregnant (NP) females at day 5 were cultured for 12 days in the presence of recombinant mouse GM-CSF (Serotec, USA) and exposed to LPS (1 µg/ml) alone, LPS+CoPPIX (50 µM) or LPS+ZnPPIX (20 µM) for 48 h. DCs were analyzed for the expression of maturity markers by flow cytometry. Maturity status is indicated by CD11c^+^CD80^+^ (**A**) and CD11c^+^MHCII^+^ cells (**B**). Statistical analysis was performed by one way ANOVA. **:p<0.01, *:p<0.05 and #:p<0.1.

**Figure 7:**
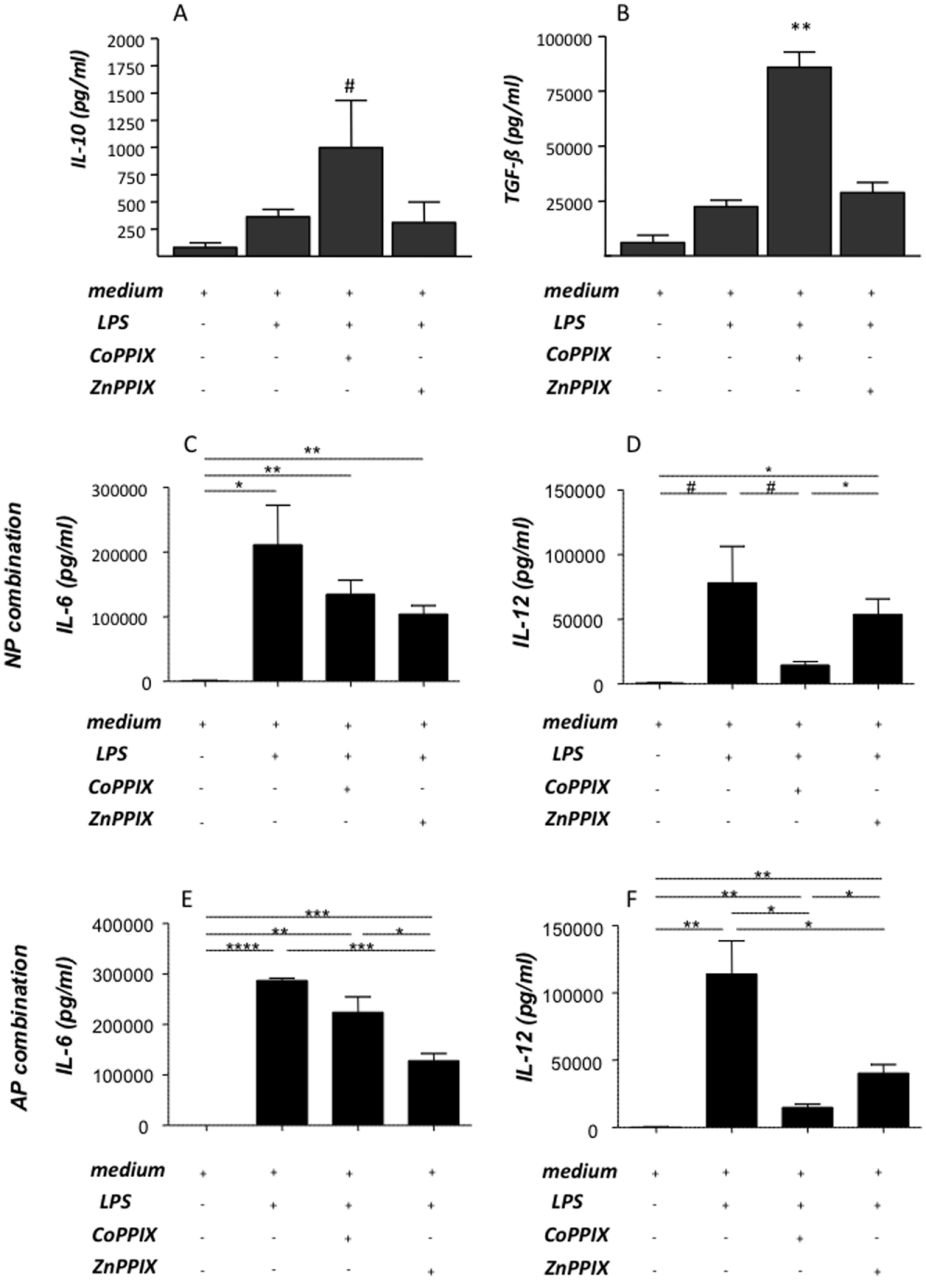
In vitro CoPPIX treatment promotes IL-10 and TGF-ß secretion by dendritic cells while diminishes their ability to secrete IL-12. **A–F** depict data obtained (mean ± SEM) in six experiments performed in duplicates or triplicates for IL-10 (pg/ml), TGF-β (pg/ml), IL-6 (pg/ml) and IL-1. In (A–D) the DCs were isolated from pregnant mice of the NO combination, while in (E–F) the DCs were obtained from animals from the AP combination. Cytokines were measured by ELISA. Statistical analysis was performed by one way ANOVA. **:p<0.01, *:p<0.05 and #:p<0.1.

We next investigated the effect of HO-1 modulation on DCs *in vitro* by exposing bone marrow-derived DCs from normal pregnant female donors to LPS added of CoPPIX or ZnPPIX. The highest number of mature DCs, as analyzed by measuring CD11c^+^CD80^+^ and CD11c^+^MHCII^+^ cells, was found in cultures in which DCs were treated with both LPS and ZnPPIX (Fig. 6A and 6B). This reinforces our previous results on HO-1 down-regulation as the trigger for DC maturation. The effect of CoPPIX, already demonstrated to up-regulate HO-1 in DCs *in vitro* [41], was hindered in LPS-treated cells as the percentage of CD11c^+^CD80^+^ and CD11c^+^MHCII^+^cells of DC treated with LPS together with CoPPIX or ZnPPIX was comparable (Fig. 6A and 6B). We next measured the secretion of anti-inflammatory cytokines, IL-10 and TGF-ß in supernatants from DCs previously cultured under different conditions. LPS alone induced DCs to secrete IL-10 and TGF-ß cytokines (Fig. 7A and 7B). IL-10 and TGF-ß secretion by LPS-treated DCs was not influenced by the addition of ZnPPIX to the cultures (Fig. 7A and 7B). Addition of CoPPIX to the DC culture, which is known to augment HO-1, led to an augmentation in the levels of secreted IL-10 and TGF-β (Fig. 7A and 7B). When analysing the levels of IL-6 and IL-12 in supernatant of cultured DCs from either NP (C and D) or AP (E and F) mice we observed that the up-regulation of HO-1 by CoPPIX leads to a significant decrease in the production of IL-6 and IL-12 by DCs from both mating combinations (Fig. 7C–F). Our results suggest that HO-1 increases LPS-induced production of anti-inflammatory cytokines in DCs, which is once again indicative of HO-1-mediated DC maturation. All together, our results clearly show that HO-1 modulation *in vitro* influences DCs in their maturation state, their function and in their cytokine secretion pattern. HO-1 augmentation is related to immature, thus tolerogenic DCs while HO-1 blockage leads to mature, functioning DCs. Maturation state of DCs have then an impact on Treg number and activity, which greatly influences pregnancy outcome.

## Discussion

Pregnancy establishment and maintenance constitutes a huge challenge for the maternal immune system as it has to on the one hand be able to combat infections and on the other hand tolerate the fetus expressing foreign paternal antigens. It has been shown that regulatory T cells (Treg) are of importance in achieving tolerance and avoiding maternal effector cells to attack fetal structures.

In the present study, we aimed to investigate whether the proven protective effect of regulatory T cells on pregnancy outcome is mediated by the enzyme Heme oxygenase-1 (HO-1) as their interplay has been already described for other pathologies. It is known that HO-1 has profound effects on reproductive steps. It affects ovulation and fertilization in mice [42] and also known to be highly expressed by trophoblast cells already at early pregnancy stages [43]. HO-1 diminution is related to murine and human pregnancy complications [27], [28], [44] while its augmentation can rescue from fetal death [27], [28]. It is known that some of the protective effects of HO-1 in pregnancy are mediated by carbon monoxide [24]. Furthermore, HO-1 micro-polymorphism in women is related to repeated miscarriage [45]. However, it was unknown whether HO-1 also exerts a protective function during pregnancy by modifying immune responses in the context of paternal antigen recognition and tolerance. To answer this question, we first took advantage of the well-established mating combinations CBA/J×BALB/c constituting a normal pregnancy pairing (NP) as well as the mating CBA/J×DBA/2J which represents an abortion-prone combination (AP), which spontaneously shows elevated abortion rates. Both combinations differ only in minor histocompability antigens in the males [29]. This model has been widely used and is well-established as a valid one to investigate mechanisms underlying either maternal tolerance towards the fetus or fetal rejection. In this particular model, the tissue protective enzyme HO-1 is lower expressed at the fetal-maternal interface from AP animals as compared to NP controls at day 14 of pregnancy [28], [33]. The different HO-1 levels seem to be due to genetic differences in the F1 tissue as DBA/2J males present lower HO-1 expression in several tissues as e.g. testicles and spleen. This is in line with other studies showing that *Hmox1* genetic ablation in parents affects placental growth as compared to wild type controls [24], [43]. Furthermore, using this model, we have been able to show that Treg are of central importance for preventing maternal T cells to attack the fetus [13]. Transfer of Treg obtained from NP animals at their day 14 of pregnancy into AP females could rescue from abortion [13]. Treg transfer was shown to be effective only when performed immediately after detection of pregnancy [13], [18] and Treg can protect male minor antigens due to linked immunosuppression [17]. Treg transfer was able to create a protective, transient micromillieu at the fetal-maternal interface characterized by high levels of LIF, TGF-β and HO-1 [18].

Recent studies have implied a critical role of HO-1 on suppressive function of Treg [46], [47]. Induction of HO-1 results in a significant up-regulation of Foxp3, TGF-ß and CTLA-4 expression in cardiac transplants and cardiac transplantation failed in *Hmox1* ^−/−^ mice [22]. In view of the obvious interplay between HO-1 and Treg in tolerance in other systems, we went ahead and studied whether HO-1 mediates the protective effect of Treg in pregnancy tolerance and the mechanisms behind. We confirmed a positive correlation among *Hmox1* and *foxp3* at mRNA levels in the fetal-maternal interface, which inversely correlates with abortion rates. Clearly high levels of HO-1 and high Treg frequencies imply normal pregnancies and low HO-1 and Treg levels correlate with abortion.

HO-1 blockage by ZnPPIX at early pregnancy stages resulted in increased abortion rates not only in AP mice but also in NP animals. This was related to slightly diminished number of Treg as reported in other models [39]. Most important, the function of Treg was affected after *in vivo* blockage of HO-1, as we observed when sorting Treg from *Foxp3^gfp^* transgenic mice [30] and studied their suppressive ability *in vitro*. Our data agree with the data by George *et al.* [41], who observed that the activity of *Hmox1* ^+/+^ Treg is compromised *in vitro* by *Hmox1* ^−/−^ DCs, an observation which could not be reproduced by other group [35], who reported no changes in the number or function of Treg in *Hmox1^−/−^* mice. However, that *Hmox1^−/−^* mice do not present different Treg levels does not necessarily exclude that HO-1 may have an effect on Treg if present in the system or may even modulate Treg function via other cells. Our data on HO-1 affecting Treg during pregnancy may further unravel a hormone-dependent effect of HO-1 on Treg, which was not deeper studied in this context.

We have found both, lower *Hmox1* and *foxp3* levels in AP animals compared to NP controls and confirmed that HO-1 blockage has negative effects on pregnancy outcome associated with diminished levels and unpaired function of Treg. Genetic ablation of *Hmox1* results in no progeny [25] which is due to intrauterine fetal death caused by an excess and free heme, thus to inadequate heme catabolism [24], while Treg absence is related to increased abortion rate but still gives progeny [14]; thus HO-1 activity seems to be more important for pregnancy outcome than the presence of Treg or Treg may work via HO-1. Therefore, our next aim was to elucidate whether the transfer of Treg can still protect from fetal rejection when HO-1 is inhibited by ZnPPIX. To achieve this goal, we transferred Treg [13] while blocking HO-1 by ZnPPIX. It has to be mentioned that, unfortunately, no *Hmox1* deficient animals are available on a CBA/J background to test the effect of permanent absence of HO-1 enzymatic activiy in this particular model of adoptive transfer. However, ZnPPIX is widely established as a highly efficient treatment to temporarily silence HO-1 [48], [49]. To ensure that HO-1 was blocked during establishment of pregnancy and implantation, three doses of ZnPPIX were employed after checking this in preliminary experiments based on the half-life of ZnPPIX [50]. ZnPPIX-mediated blockage of HO-1 impressible resulted in total abrogation of Treg's protective effect on pregnancy. Treg-transferred, ZnPPIX-treated animals showed higher abortion rates compared to the Treg-transferred, PBS-treated controls and these abortion rates. This is very indicative of HO-1 being necessary for Treg to protect from fetal rejection. Our data further position HO-1 in a different, unknown level and confirm that HO-1 is more relevant than Treg in gestation, yet it allows Treg to be functional during pregnancy. Our data on elevated placental levels of HO-1 after Treg transfer [18] further suggest a bidirectional communication between these two important players and a mechanism of self-regulation by Treg: the more they are generated the more HO-1 is produced which in turns allows Treg to protect allo-fetuses against abortions.

We next examined the mechanisms as to how HO-1 may direct Treg to be protective. It has been recently reported that HO-1 induction in CD4^+^CD25^−^ T cells does not transform these cells into cells with immunoregulatory or immunosuppressive function [51]. In agreement with this, Treg of *Hmox1* ^−/−^ mice seem to be fully functional [35]. It is therefore not probable that the effects we observed after HO-1 blockage are due to direct modification of Treg, which express HO-1 [51] or to modifications in the function of effector cells. An indirect action of HO-1 on Treg immunosuppression activity by its expression and regulation in DCs has been shown in several models of immune pathologies [52]–[54]. In fact, antigen-presenting cells (APCs), and among these, DCs, express HO-1 and their levels are regulated by application of CoPPIX or ZnPPIX both *in vitro* and *in vivo* [36,reviewed in 40]. HO-1 induction leads to immature DCs and provokes loss of their immunogenicity [35]. Moreover, HO-1 expression by DCs is necessary for CD4^+^CD25^+^ Treg to exert their immunoregulatory activity. As stated by Blancou and Anegon in their Editorial Comment, HO-1 may be dispensable for Tregs to exert their effect but profoundly affects DC function which indirectly influences Treg [55]. We therefore concentrated on the effect of HO-1 regulation on DC maturation during pregnancy. We have also chosen DCs as they can direct the immune response towards rejection or tolerance depending on their maturation state [55]. The crucial role of DCs for pregnancy has been recently demonstrated by Plaks *et al*. [56]. It was also reported that DCs known to be tolerogenic after exposure to Gal-1 [57], [58] can prevent abortion caused by sonic stress [38]. No reports are available addressing the role of tolerogenic DCs in a more physiological model, e.g. models of spontaneous abortions or normally progressing pregnancies. Besides the direct evidence that HO-1 renders APCs into tolerogenic cells [36], [41], HO-1 has also been reported to marginally protect allo-grafts by inhibiting the immunogenicity of donor-derived DCs *in vivo* [59], [60]. Here, we clearly show that HO-1 manipulation during pregnancy greatly interferes with *in vivo* DC maturation. Isolated CD11c^+^ DCs from ZnPPIX-treated animals foster the proliferation of responder cells from wild type pregnant animals when compared to the co-cultures in which isolated DCs from CoPPIX-treated animals were used. As DCs were obtained from CoPPIX- or ZnPPIX-treated pregnant animals, these results are supposed to mirror the *in vivo* situation. It is therefore tempting to postulate that low HO-1 levels allow DC to mature and therefore stimulate maternal effector cells to proliferate, which finally results in attack and clearance of cells expressing paternal antigens, hence fetal rejection or abortion. This would be the case for either individuals genetically expressing lower HO-1 levels, e.g. *Hmox1* ^+/−^ or *Hmox1* ^−/−^ animals or AP animals. High HO-1 levels, like spontaneously occurring in NP animals, retain DCs in immature state that allows Treg to suppress effector T cells from fetal attack. Our data are in line with interesting data revealing that exposure of murine DCs to serum of pregnant mice diminish their capacity to induce production of Th1 cytokines and allogenic T cell proliferation [61]. HO-1 hindrance effect on maturation of DCs that have seen paternal antigens may inhibit pro-inflammatory and allogeneic immune responses while preserving IL-10 production, results which agree with the findings reported in other systems [36]. The higher proliferation rate of effector cells in contact with ZnPPIX-treated DCs was accompanied by lower levels of cells with suppressor or regulatory phenotype. HO-1 systemic augmentation seems not only to block DC maturation but also to favour Foxp3 expression in CD4^+^ cells. Drug-based inhibition of HO-1 was shown to affect maternal and fetal hemodynamic, leading to loss of maternal blood and pooling of fetal blood in the labyrinthine areas of the placenta [62] as well as to elevated levels of soluble endoglin and sFlt-1 [62]. This might compromise fetal survival. However, HO-1 blockage at early stages of pregnancy as we do here should not modify much the angiogenesis of placenta. Trophoblasts also express HO-1, being its expression also regulable by CoPPIX and ZnPPIX [26]. It is probably that HO-1 expression in trophoblasts also contributes to Treg number/action. This was however, not analyzed in this context.

To gain a further insight into HO-1 modulation of DCs, we next isolated bone marrow-derived DCs and treated them with LPS and either CoPPIX or ZnPPIX. We analyzed DCs for the expression of maturity markers as well as for their cytokine secretion profile. DCs that received LPS together with ZnPPIX contained the highest proportion of mature DCs (mDCs) as indicated by higher percentages of CD11c^+^CD80^+^ and CD11c^+^MHCII^+^ cells. CoPPIX addition to the culture did not significantly modify the expression of maturation markers. Thus, DCs expressing less HO-1 are more mature. Absolute confirmation of the importance of HO-1 expression in DCs for their maturation was obtained when analyzing the maturity state of DCs obtained from *Hmox1* ^+/+^, *Hmox1* ^+/−^ and *Hmox1* ^−/−^ females. Partial and total absence of HO-1 leaded to more expression of maturity markers, regardless of LPS addition to the culture. These data implicate once again HO-1 as one of the determining factors in the maturation of DCs. We further observed that the addition of CoPPIX to DC cultures, which did not affect the levels of maturity markers, lead however to augmented secretion of the anti-inflammatory cytokines, IL-10 and TGF-ß. Similarly, IL-12 production by DCs was diminished if the cells were treated with CoPPIX. Similar results have been reported elsewhere in other models through HO-1 induction [63], [64]. Our data reveals HO-1 as a strong modulator of DC maturity. Additionally, DCs provide a direct link between HO-1 and Treg as production of IL-10 and TGF-ß are known to be important for the generation and function of Treg subsets in their tolerance quest [65], [66] and of great importance for pregnancy. It has to be stated, however, that pregnancy does not generate a strong allo-response, which is logical as the allo-response generated during gestation has to protect and not reject the conceptus. Thus, the changes in the MHC levels are small. Linking these observed changes in DC phenotype and function with altered pregnancy outcomes caused by HO-1 inhibitor treatments is speculative and further, complementary mechanisms may count for that.

Understanding the mechanisms as to how Treg function at the fetal-maternal interface is of paramount importance in explaining how fetal tolerance by the maternal immune system is achieved. Here, we show that blocking HO-1 abrogates the protective effect of Treg on pregnancy outcome. This is linked to the ability of HO-1 to influence the maturity state of DCs. HO-1 augmentation favours the generation of immature DCs which in turn supports the generation and proliferation of Treg, thus fetal tolerance. HO-1 blockage favours DC maturation, which results in activation of effector cells, which can not longer be controlled by Treg and then attack fetal structures, leading to fetal rejection. Our data underline the importance of HO-1 on pregnancy outcome and show its effect of both, maturation of DC and Treg function.

## Supporting Information

Table S1 **Sequences for primers and probes.**
(DOCX)

Figure S1 **Levels of Hmox1 mRNA in tissues of BALB/c versus DBA/2J male mice.**
(DOCX)

Figure S2 **Levels of CD4+Foxp3+IL-10+ cells in blood of Foxp3.GFP mice treated with PBS (controls) or ZnPPIX.**
(DOCX)
