# Supplementary material for: Blockage of Heme Oxygenase-1 Abrogates the Protective Effect of Regulatory T Cells on Murine Pregnancy and Promotes the Maturation of Dendritic Cells
Source: PLoS One. 2012 Aug 10;7(8):e42301. doi: 10.1371/journal.pone.0042301 (PMC3416808; doi:10.1371/journal.pone.0042301)
Supplement: Table S1 — Sequences for primers and probes. (DOCX) [file pone.0042301.s001.docx]

Schumacher et al.

Blockage of Heme Oxygenase-1 abrogates the protective effect of regulatory T cells on pregnancy and promotes the maturation of dendritic cells

Supplementary material

Table S1: ***Sequences for primers and probes***

|  | **Primer name** | **Primer sequence 5’- 3’** |
| --- | --- | --- |
| 1 | *foxp3*  Probe-FAM-TAMRA labelled | **F -** CCC AGG AAA GAC AGC AAC CCT  **R -** TTC TCA CAA CCA GGC CAC TTG  - ATC CTA CCC ACT GCT GGC AAA TGG AGT C |
| 2 | *Hmox1*  Probe-FAM-TAMRA labelled | **F-** CAG AAG AGG CTA AGA CCG CCT T  **R-** TCT GGT CTT TGT GTT CCT CTG TCA  - TGC TCA ACA TTG AGC TGT TTG AGG AGC TG |
| 3 | *ß-actin*  Probe-FAM-TAMRA labelled | **F -** GCT TCT TTG CAG CTC CTT CGT T  **R -** GTT GTC GAC GAC CAG CGC  - CAG CCT TCC TTC TTG GGT ATG GAA TCC T |
