# Supplementary material for: Blockage of Heme Oxygenase-1 Abrogates the Protective Effect of Regulatory T Cells on Murine Pregnancy and Promotes the Maturation of Dendritic Cells
Source: PLoS One. 2012 Aug 10;7(8):e42301. doi: 10.1371/journal.pone.0042301 (PMC3416808; doi:10.1371/journal.pone.0042301)
Supplement: Figure S1 — Levels of Hmox1 mRNA in tissues of BALB/c versus DBA/2J male mice. (DOCX) [file pone.0042301.s002.docx]

Schumacher et al.

Blockage of Heme Oxygenase-1 abrogates the protective effect of regulatory T cells on pregnancy and promotes the maturation of dendritic cells

Supplementary material

Figure S1: ***Levels of Hmox1 mRNA in thymus of BALB/c males (white) versus DBA/2J males (grey)***

n=6

n=4

0.001

0.002

0.003

0.004

0.005

0.006

***Hmox1 mRNA 2(^-ΔCT^)***

#

#: p>0.05 and <0.1

***Levels of Hmox1 mRNA in spleen of BALB/c males (white) versus DBA/2J males (grey)***

*p>0.05

n=6

n=5

0.04

0.06

0.08

0.10

0.12

0.14

0.16

*

***Hmox1 mRNA 2(^-ΔCT^)***
