# Supplementary material for: Blockage of Heme Oxygenase-1 Abrogates the Protective Effect of Regulatory T Cells on Murine Pregnancy and Promotes the Maturation of Dendritic Cells
Source: PLoS One. 2012 Aug 10;7(8):e42301. doi: 10.1371/journal.pone.0042301 (PMC3416808; doi:10.1371/journal.pone.0042301)
Supplement: Figure S2 — Levels of CD4+Foxp3+IL-10+ cells in blood of Foxp3.GFP mice treated with PBS (controls) or ZnPPIX. (DOCX) [file pone.0042301.s003.docx]

Schumacher et al.

Blockage of Heme Oxygenase-1 abrogates the protective effect of regulatory T cells on pregnancy and promotes the maturation of dendritic cells

Supplementary material

Figure S2: ***Levels of CD4+Foxp3+IL-10+ cells in blood of Foxp3.GFP mice treated with PBS (controls) or ZnPPIX***

**
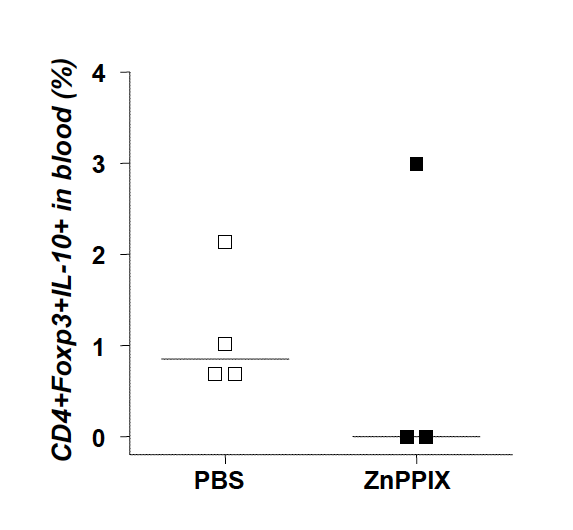
**
